# A compact multi-pixel superconducting nanowire single-photon detector array supporting gigabit space-to-ground communications

**DOI:** 10.1038/s41377-023-01374-1

**Published:** 2024-01-22

**Authors:** Hao Hao, Qing-Yuan Zhao, Yang-Hui Huang, Jie Deng, Fan Yang, Sai-Ying Ru, Zhen Liu, Chao Wan, Hao Liu, Zhi-Jian Li, Hua-Bing Wang, Xue-Cou Tu, La-Bao Zhang, Xiao-Qing Jia, Xing-Long Wu, Jian Chen, Lin Kang, Pei-Heng Wu

**Affiliations:** 1 Research Institute of Superconductor Electronics (RISE), School of Electronic Science and Engineering, Nanjing University, Nanjing, Jiangsu 210023 China; 2 Purple Mountain Laboratories, Nanjing, Jiangsu 211111 China; 3 Hefei National Laboratory, Hefei, Anhui 230088 China; 4 National Laboratory of Solid State Microstructures and Department of Physics, Nanjing University, Nanjing, 210023 China

**Keywords:** Fibre optics and optical communications, Single photons and quantum effects, Photonic devices

## Abstract

Classical and quantum space-to-ground communications necessitate highly sensitive receivers capable of extracting information from modulated photons to extend the communication distance from near-earth orbits to deep space explorations. To achieve gigabit data rates while mitigating strong background noise photons and beam drift in a highly attenuated free-space channel, a comprehensive design of a multi-functional detector is indispensable. In this study, we present an innovative compact multi-pixel superconducting nanowire single-photon detector array that integrates near-unity detection efficiency (91.6%), high photon counting rate (1.61 Gcps), large dynamic range for resolving different photon numbers (1–24), and four-quadrant position sensing function all within one device. Furthermore, we have constructed a communication testbed to validate the advantages offered by such an architecture. Through 8-PPM (pulse position modulation) format communication experiments, we have achieved an impressive maximum data rate of 1.5 Gbps, demonstrating sensitivities surpassing previous benchmarks at respective speeds. By incorporating photon number information into error correction codes, the receiver can tolerate maximum background noise levels equivalent to 0.8 photons/slot at a data rate of 120 Mbps—showcasing a great potential for daylight operation scenarios. Additionally, preliminary beam tracking tests were conducted through open-loop scanning techniques, which revealed clear quantitative dependence indicating sensitivity variations based on beam location. Based on the device characterizations and communication results, we anticipate that this device architecture, along with its corresponding signal processing and coding techniques, will be applicable in future space-to-ground communication tasks.

## Introduction

Human and robotic explorations from deep space extend human presence beyond low Earth orbit, disclose the mysteries of the universe, and search inhabited planets. To support these ambitious space missions, enhanced telecommunication capacity is critically required for transferring massive amounts of data generated by advanced scientific instruments. Space-to-ground laser communication has superior high-speed data rates^1^, which has been demonstrated to be a revolution in space communications on Earth, between constellation satellites, and from the Moon^2^. The lunar laser communication demonstration (LLCD) could be considered a remarkable step towards deep-space laser communication, which achieved 622 Mbps downlink speed, an order of magnitude faster than the best Ka-band radio system flown to the moon (100 Mb/s) on the Lunar Reconnaissance Orbiter in 2009^3^. Establishing laser communications in deep-space scenarios would be one of the next milestones. NASA’s Psyche Mission hosts a flight laser transceiver for link demonstrations extending from 0.1 to farther than 2 astronomical units (AU)^4^. Likewise, the European Space Agency (ESA) is planning a deep-space optical communications system technology demonstration from the Sun–Earth Lagrange (L5) point, hosted by their Space Weather Mission spacecraft^5^.

Technical challenges still remain in deep-space optical communications. A ground-based optical receiver has to be sufficiently sensitive to compensate for huge space signal loss, reduce the aperture size of the downlink receiver telescope, or mitigate the power budget of the space load. Although the recent development of coherent receivers has shown improved sensitivity^6^, photon-counting-based receiver architecture has competitive efficiency. Combined with PPM and single-photon detectors (SPDs), the receiver can have a sensitivity of a few bits per received photon^7^. Therefore, high-performance single-photon detectors are the key elements in building the ground receiver in the past and future deep space communication missions mentioned previously. For quantum space-to-ground communications, the demands for high-performance detectors are even more pronounced due to the significantly weaker quantum state compared to classical signals^8–10^. In general, from the perspective of the ground-based optical receiver, both classical and quantum communications rely on the sensitivity and speed of the detector to determine the ultimate distance and data rate.

Among several existing SPDs, the superconducting nanowire single-photon detector (SNSPD), which has been used in LLCD^11^, is preferred to PPM formatted communication due to its high efficiency, low timing jitter, and free-running operation mode. The SNSPD’s performance has improved significantly in recent years^12^. The system detection efficiency (SDE) has increased to higher than 90%^13–17^, the timing jitter has reduced to less than 10 ps^18^, the dark count rates (DCRs) have reduced to below 6 × 10^6^/s^19^, and the count rate has increased up to 1.5 GHz with array structures^20–22^. Due to its exceptional performance, the SNSPD has found applications in various space-to-ground communications or laboratory experiments, as demonstrated in the following studies. A communication rate of 781 Mbit/s was achieved in^23^. The LLCD accomplished a downlink rate of 622 Mbit/s^24,25^. In addition, a multi-element SNSPD was utilized to achieve a communication data rate of 1.25 Gbit/s^26^. In the ongoing experiment of NASA’s deep-space optical communication project, the ground receiver uses a 64-pixel SNSPD array^27^. Although the state-of-the-art timing jitter of an SNSPD can support a PPM slot frequency of above 10 GHz, the reset time is the dominant bottleneck due to two tradeoffs. The first tradeoff is between the detector’s active area and the reset time. Increasing the active area can improve the optical coupling and the system detection efficiencies. However, a long nanowire is required to cover this active area, giving a large kinetic inductance that slows down the bias current recharging^28^. Although using an array of SNSPDs, which operates multiple pixels in parallel, can shorten the average reset time^20–22^, a second tradeoff between the readout complexity and the array size is then introduced. Individual reading of a large number of pixels increases the thermal load at cryogenic temperature and makes the communication receiver hardware more complicated, including digitization, synchronization, and decoding.

In this study, we demonstrate a compact architecture of a multi-pixel SNSPD array arranged into a four-quadrant (4-QD) geometry with four output leads. This 4-QD detector effectively addresses the tradeoffs mentioned earlier and integrates essential features required for deep space communications, including high detection efficiency, elevated counting rate, photon-number resolving capability, and position-resolving capability. These advantages are combined in a compact design, which is optimized for high-speed and energy-efficient free-space optical communications. Meanwhile, a specialized signal processing technique is introduced to effectively extract photon information from pile-up detection pulses. The detector has a device detection efficiency of 91.6%. The minimum timing jitters of the detector are 78 ps (single photon) and 21 ps (six photons). Instead of using a single nanowire, each quadrant of the detector adopts a serial assembly, which increases the total photon counting rate to 1.6 Gcps and gives a photon number resolving (PNR) capability from 1 to 24 photons. Considering only four outputs are used, this architecture is much more compact than conventional high-speed multi-pixel SNSPDs.

These advantages meet the challenges in long-haul ground-to-space communication. Based on this high-performance detector, we built a PPM communication testbed. At the 8-PPM format, the receiver sensitivities were 1.47, 1.76, 3.40, and 7.41 photons/bit at bit rates of 480 Mbps, 800 Mbps, 1.2 Gbps, and 1.5 Gbps, respectively. The PNR ability overcomes the 1-bit dynamic range bottleneck in conventional SNSPDs and gives the receiver strong immunity to the background noise photons. Experimentally, the receiver maintained an error-free link when the background noise increased to 0.8 photons per PPM slot with a communication rate of 120 Mbps, supporting daytime operations. Additionally, the 4-QD offers position sensitivity in a photon counting mode, which is able to track the beam position to maintain the receiver at its best sensitivity. We envision that these demonstrations would be interesting for special space-to-ground laser communication missions.

## Results

### Detector architecture and characterization

**Fig. 1:**
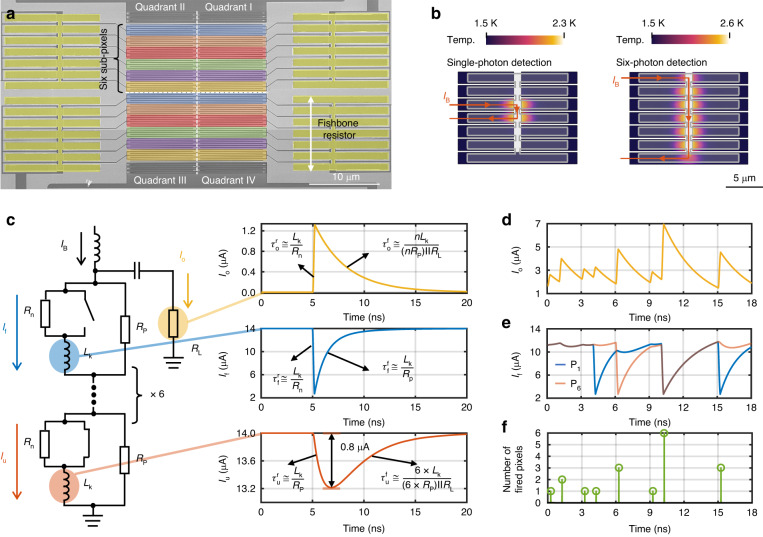
**Detector architecture. a** Scanning electron microscope (SEM) image of the 4-QD. **b** Simulations of the temperature distribution of the niobium-nitride (NbN) surface below the “fishbone” resistor when the third sub-pixel fires and all six sub-pixels fire. **c** The equivalent circuit of the serial SNSPD. The yellow, blue, and red waveforms correspond to the transient currents of the output ${I}_{\text{o}}$, the fired sub-pixel ${I}_{\text{f}}$, and the unfired sub-pixel ${I}_{\text{u}}$ separately at single photon detection. ${\tau }_{\text{o}}^{\text{r}}$ and ${\tau }_{\text{o}}^{\text{f}}$ are the leading edge time and recovery time of the output pulse respectively. ${\tau }_{\text{f}}^{\text{r}}$ and ${\tau }_{\text{f}}^{\text{f}}$ are the leading edge time and recovery time of the current through the fired pixel respectively. ${\tau }_{\text{u}}^{\text{r}}$ and ${\tau }_{\text{u}}^{\text{f}}$ are the leading edge time and recovery time of the current through the unfired pixel respectively. **d** The transient currents of the output ${I}_{\text{o}}$ at a high counting rate. **e** The corresponding transient currents of P_1_ (sub-pixel 1) and P_6_ (sub-pixel 6). **f** The number of fired pixels extracted from the detector’s output pulse

Figure 1a shows the scanning electron microscope picture of the device. It is a 2 × 2 array, which can give a position-sensing capability by operating it as a photon-counting 4-QD. The array is referred to as a 4-QD in the remaining part of this article. For each quadrant, the nanowire is divided into six pixels, referred to as sub-pixels. The detector fabrication process is given in the supplementary materials, and the detector parameters are given in the methods. Each sub-pixel is shunted by a neighboring resistor, designed in a fishbone geometry as shown in Fig. 1b. Compared with a stripline resistor, the fishbone resistor speeds up thermal cooling, preventing the nanowire from latching at a high counting rate. The serial architecture, which was invented originally for obtaining PNR capability, was found to offer a high photon counting rate in our previous work^29^. Figure 1c–f give simulation results by using a nanowire SPICE model^30^. As shown in Fig. 1c, ${I}_{\text{B}}$ is the bias current. ${I}_{\text{f}}$ and ${I}_{\text{u}}$ are the current through the fired and unfired sub-pixel respectively. ${I}_{\text{o}}$ is the output current. ${R}_{\text{n}}$ is the resistance of the normal state of each sub-pixel. ${L}_{\text{k}}$ is the kinetic inductance for each sub-pixel, and ${R}_{\text{p}}$ is the shunted resistance. When one sub-pixel fires, its recovery time constant is ${\tau }_{\text{f}}^{\text{f}}\,\cong \,{L}_{\text{k}}/{R}_{\text{p}}$. As each sub-pixel is shunt by a resistor, when a pixel fires, the bias current can still go to the rest pixels through the resistor. Therefore, the six sub-pixels can detect photons almost successively. It is noticeable that although the output pulse still shows a falling edge limited by the total inductance, it does not manifest a slow counting rate for all sub-pixels. As shown in Fig. 1d, e, when several photons incident on different sub-pixels in sequence, their outputs pile up while the unfired sub-pixels still maintain in the standby state with a slightly reduced bias current. The pile-up effect brings difficulties in extracting photon arrival times and photon numbers, which can be solved by using a digital signal processing method given in the following section. The number of fired sub-pixels can be extracted from the amplitude of the output pulse, as shown in Fig. 1f.

Compared to the SNSPD array in which pixels are biased and read out by separate circuits, the serial SNSPD array features all pixels being biased and read out by a common line, thereby offering a compact design for the construction of a communication receiver and reducing the thermal load of the cryostat. However, a trade-off between the pulse amplitude ${V}_{\text{amp}}$ and the sub-pixel number *n* exists. The amplitude of single-photon detection pulses can be determined as Eq. 1: (refer to Supplementary materials for detailed derivation).

1 $${V}_{{\rm{amp}}}=\left({I}_{{\rm{B}}}-{I}_{{\rm{min}}}\right)\,\cdot\, \frac{{R}_{{\rm{p}}}}{\left(1+n\,\cdot\, \frac{{R}_{{\rm{P}}}}{{R}_{{\rm{L}}}}\right)}$$

**Fig. 2:**
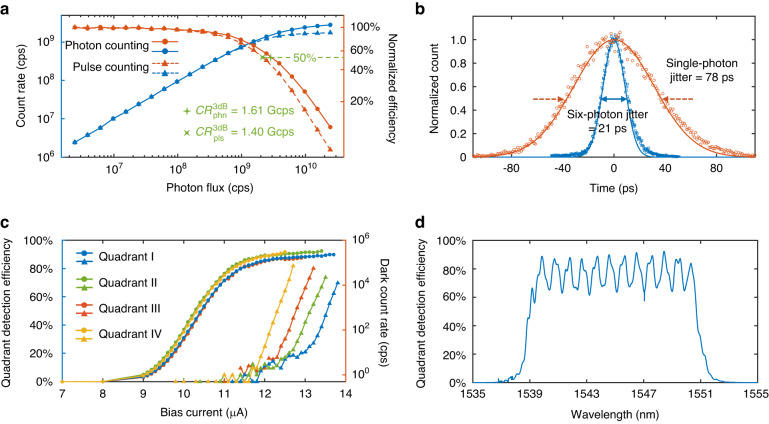
**Detector performance. a** Dependences of the whole detector’s photon and pulse counting rates (blue curves) on the incident photon flux are plotted on the left *y*-axis. The corresponding normalized detection efficiencies (red curves) are plotted on the right *y*-axis. **b** Timing jitters of single-photon and six-photon responses for the first quadrant. **c** Detector efficiency and dark counts of each quadrant vs. bias current. The dot lines show the efficiencies and the triangle lines show the dark counts. **d** Response spectrum of the first quadrant, measured at 12.6 μA bias current

*I*_min_ is the minimum current at which the current through the fired nanowire drops. As *n* increases, ${V}_{\text{amp}}$ drops by a factor of ${1}+{n}\cdot{R}_{\text{P}}/{R}_{\text{L}}$, which ${R}_{\text{P}}/{R}_{{\rm{L}}}=$ 1.12 and *n* = 6 in our design. The reduction of the pulse amplitude leads to a decrease in the signal-to-noise ratio, thereby compromising the accuracy of timing jitter and photon number discrimination. Although sub-pixels are connected in serial, there is only minor current crosstalk due to the shunt of a fired pixel, as shown in Fig. 1c. Furthermore. a saturated detection efficiency at a high bias current exhibited by the detector (Fig. 2c) ensures that even this slight reduction in bias current does not significantly affect photon counting efficiency.

The total counting rate of the whole detector was characterized, and results are shown in Fig. 2a. As each quadrant can output pulse height proportional to the incident photon number, as shown in Fig. 6a, and the coherent laser pulse has a Poisson distribution for photon numbers, there are two metrics to characterize the counting speed, which are the photon counting rate ${{CR}}_{\text{phn}}$ and pulse counting rate ${{CR}}_{\text{pls}}$. The ${{CR}}_{\text{pls}}$ only counts the number of output pulses, while the ${{CR}}_{\text{phn}}$ counts the photon numbers represented by each output pulse (For instance, the ${{CR}}_{\text{pls}}$ in Fig. 6a is 4, and the ${{CR}}_{\text{phn}}$ is 7). The laser repetition rate was set as 1 GHz, and the attenuation was swept to vary the incident photon flux intensity. At strong attenuations, the mean photon number per pulse *µ* is much less than one. Therefore, the incident photons are almost in pure single-photon events, and ${{CR}}_{\text{phn}}$ is almost equal to ${{CR}}_{\text{pls}}$. As *µ* increases to have more multi-photon events, ${{CR}}_{\text{phn}}$ is higher than ${{CR}}_{\text{pls}}$. When the photon and pulse detection efficiencies drop by 3 dB, the corresponding counting rates are ${{CR}}_{{\rm{phn}}}^{3\text{dB}}$ = 1.61 Gcps and ${{CR}}_{{\rm{pls}}}^{3\text{dB}}$ = 1.40 Gcps. The reason for the counting rates being higher than the laser repetition is that the results are the total counting rates from four quadrants. In a communication system, ${{CR}}_{\text{pls}}$ determines the maximum repetition rate of the PPM symbols, and ${{CR}}_{\text{phn}}$ benefits the forward error correction (FEC) performance for a more precise calculation of the likelihood ratio (LLR).

The serial architecture comes at a cost of a weaker output pulse amplitude, which then worsens the timing jitter in the presence of voltage noise. To overcome this problem, cryogenic amplifiers placed at 4.2 K were used to compensate for the signal loss. Because the serial detector has PNR capability and the pulse amplitude is proportional to the number of detected photons, the timing jitter for single-photon detections is worse than the timing jitter for multi-photon detections. The timing jitter of the first quadrant is shown in Fig. 2b as an example. The timing jitters of the single-photon and six-photon response output pulses are 78 ps and 21 ps, respectively (The jitter data of the other three quadrants and measurement process are given in the Supplementary material). Although these values are below the state-of-art values, they are already small enough to support PPM slot frequencies up to 10 GHz, guaranteeing communication bit rates up to Gbps.

This 4-QD architecture does not scarify the detection efficiency since the nanowires are still placed in a high filling factor. By integrating with an optical cavity to obtain high light absorption, the detection efficiencies of each quadrant, characterized by a flood illumination, are 89.4%, 91.0%, 92.6%, and 93.4%, respectively. The average detection efficiency is 91.6% (the detailed process of efficiency measurements is discussed in the supplementary materials). As shown in Fig. 2c, the dependence of the detection efficiency on bias current shows a noticeable saturation plateau, indicating that the internal quantum efficiency of the nanowire is near unity. Moreover, since the efficiency is independent of the bias current at the saturation plateau, the serial architecture can perform high counting rates with less reduction of the total efficiency, agreeing with the measured photon counting results. Figure 2d shows the response spectrum of the first quadrant detector. The bandwidth is narrowed to 12 nm by cryogenic filters for suppressing background noise photons. Details of the filters and their characterizations are given in the supplementary materials. The spectrum has a periodic oscillation, which is caused by the interference in the 300 μm-thick silicon substrate at back illumination^31^. In the communication experiments where the light was focused, such interference was not observed.

### Communication demonstration

#### Communication testbed

**Fig. 3:**
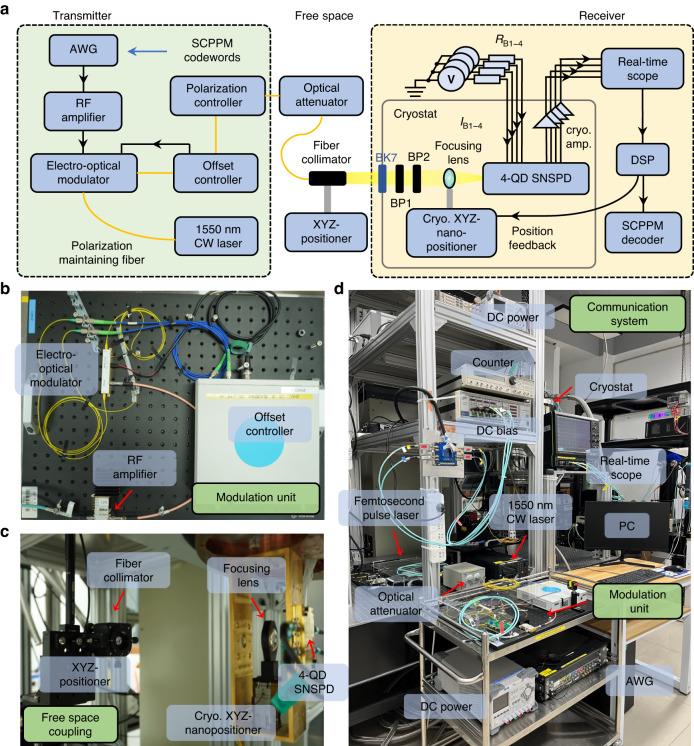
**Communication setup. a** The setup diagram for the communication testbed. The black line is the coaxial line, and the yellow line is the fiber. The 4-QD SNSPD and the nanopositioner holding the focusing lens are mounted at 1.5 K. The cryo-amplifiers and optical filters are mounted at 4.2 K. **b** The modulation unit. **c** The free space coupling system. **d** The entire communication system

We built a communication testbed based on the 4-QD. The communication system setup consists of a transmitter, a free-space channel, and a receiver, which are shown in Fig. 3a. A high-speed arbitrary waveform generator with a sampling rate of 65 G samples/sec was used for generating coded PPM electrical pulses at different bit rates. Then, the electrical pulses were amplified and input to an electro-optical modulator (EOM) for generating optical pulses. A continuous laser at a wavelength of 1550 nm was used as the seed source for the EOM. A bias offset controller was used to stabilize the EOM’s DC offset to maximize its extinction ratio, which was 40 dB at maximum. A polarization controller was used to tune the light’s polarization state to maximize the detector’s detection efficiency. Then, an optical attenuator was used to adjust the power to weak signals to simulate the loss in deep space scenarios.

Furthermore, the attenuated optical signal was collimated into free space at room temperature. The collimator was loaded on the XYZ-positioner. An aspheric lens (NA = 0.54) was loaded on the cryogenic XYZ-nanopositioner to focus the light onto the detector. The light passed through a coated BK7 glass window, a 1750 nm band-pass filter with a bandwidth of 500 nm, and a 1550 nm band-pass filter with a bandwidth of 12 nm. These two filters were mounted at 4.2 K to remove the background noise photons from thermal radiation. Additionally, four cryogenic amplifiers were mounted at 4.2 K to individually amplify the four quadrants. The detection pulses were acquired by a real-time oscilloscope with a maximum sample rate of 40 G samples/sec and then processed offline. This processing involved a series of signal pre-processing steps and the serially concatenated pulse-position modulation (SCPPM) forward error correction^32^. The system photos are shown in Fig. 3b–d to clarify how the communication system works.

In the communication experiments, the light spot was focused on the device. To utilize the full performance of the detector in the communication experiments, the spot diameter was defocused to 29.6 μm ($1/{\text{e}}^{2}$ width). This spot size was a little larger than the entire area of the nanowire so that all the nanowires could detect photons more evenly, resulting in a coupling efficiency of 70.7%. As a result, the free-space coupling system detection efficiency was reduced to 52.5%, including transmission losses from the window and filters, as shown in Table 1. A beam shaper could be used to adjust the spot into the shape of the detector for higher coupling efficiency. By reducing the diameter of the light spot to 17.8 μm, the system detection efficiency could be maximized to 72.7%. Details of the characterizations of the system efficiency can be found in the supplementary materials.

**Table 1:** Individual efficiencies in the free-space coupled communication system

Window transmission	Filters transmission	Focusing lens transmission	Spot coupling efficiency	System detection efficiency
99.8%	81.7%	99.4%	70.7%	52.5%

#### Digital signal process

**Fig. 4:**
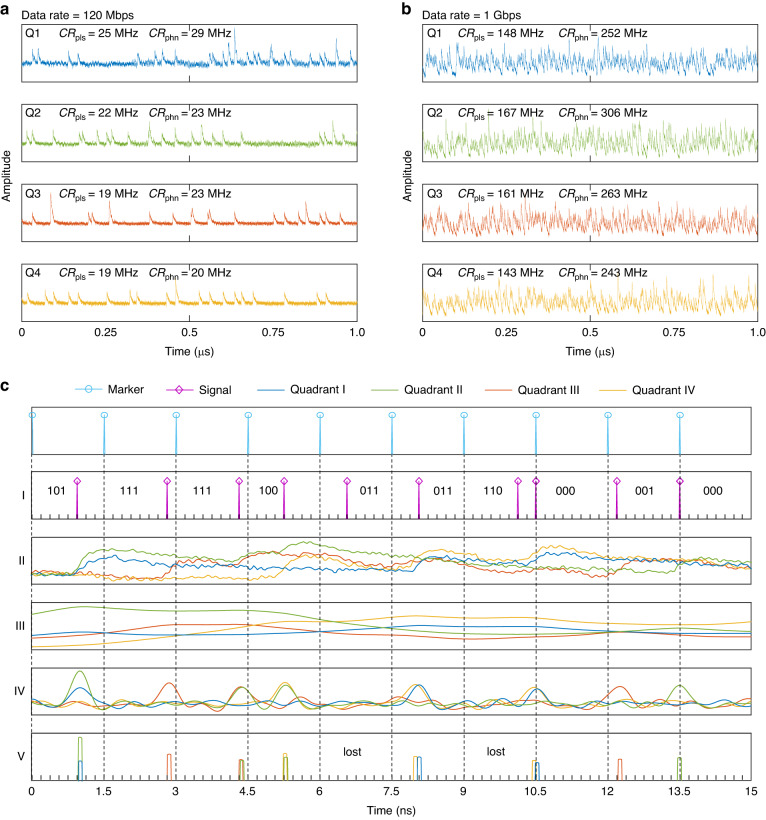
**Signal pre-processing**. Output waveforms of the 4-QD working at communication data rates of 120 Mbps (**a**) and 1 Gbps (**b**). **c**. Illustrations of signal pre-processing steps, in which the PPM format has an order of 8 with a slot width of 187.5 ps, resulting in a bit rate of 1 Gbps. I: Encoded PPM pulses; II: Four quadrants’ detection pulses; III: Outputs after matched filter; IV: Outputs after deconvolution; V: Signals extracted after peak searching

The detection pulses at low and high data rates are shown in Fig. 4a, b respectively. The pulses pile up at high data rates, similar to the inter-symbol interference problem in conventional communication systems. Although this is an evidence of fast photon counting, it is difficult for the decoder to get the correct photon information. A signal pre-processing strategy, including matched filter, deconvolution, and peak searching, was introduced to overcome this problem. An example is shown in Fig. 4c, and its details are given in the method section. The impact of signal processing techniques on the signal-to-noise ratio (SNR) and timing jitter of the output pulses from the detector has been extensively discussed in our previous research^33^. After the pre-processing, photon arrival times and detection photon numbers were extracted, which were input to the SCPPM decoder for correcting errors. At a lower data rate, such as what are shown in Fig. 4a, the output pulses are effectively separated, allowing for direct extraction of photon information. However, as the communication rate increases, the pile-up effect becomes more pronounced and leads to a higher bit error rate (BER) unless signal processing methods are employed.

#### Communication results

**Fig. 5:**
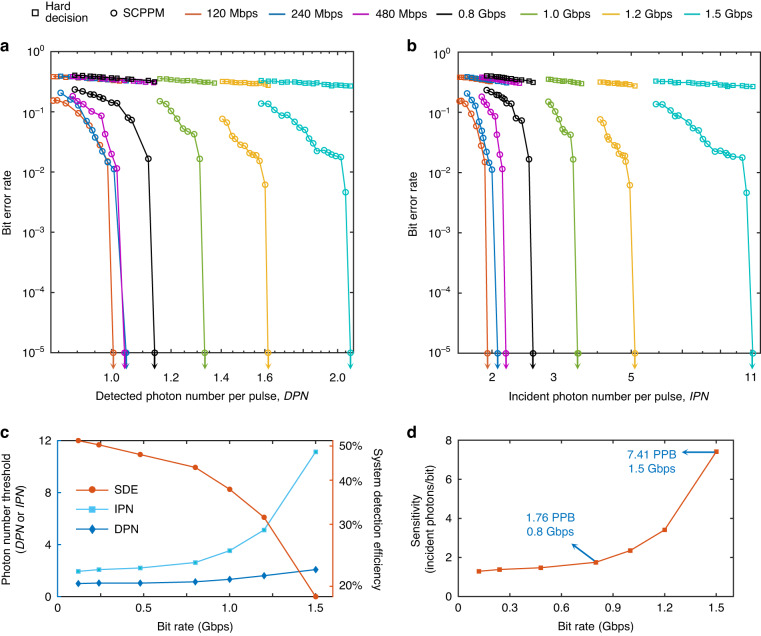
**Communication results**. BERs vs. detected (**a**) and incident (**b**) photon numbers at different bit rates. Arrows indicate the photon number thresholds where BER drops to zero for 10^5^ random bits. **c** Dependences of the photon number thresholds of *DPN*, *IPN*, and the system detection efficiency on bit rate. **d** Dependence of the sensitivity on bit rate

Based on the communication setup above, the sensitivity of the receiver prototype at different bit rates was characterized. Compared to other modulation formats, PPM emerges as an energy-efficient modulation technique for deep-space communication^34–36^. The coding scheme utilized the 1/2-rate SCPPM, which was successfully demonstrated in the LLCD. The selection of the 8-order PPM format was based on considerations of the detector’s reset time and timing jitter to maximize the bit rate. A comprehensive discussion of the modulation format can be found in the supplementary materials. The bit rates were varied from 120 Mbps to 1.5 Gbps (channel bit rates before FEC were from 240 Mbps to 3 Gbps) by adjusting the slot width. No dead time slot was inserted between the adjacent PPM symbols. The number of sending bits was ${10}^{5}$. The measured BERs at different bit rates ${R}_{\text{PPM}}$ are shown in Fig. 5a, b. The detected photon number per pulse (*DPN*) and the incident photon number per pulse (*IPN*) are calibrated for the *x*-axes in Fig. 5a, b, respectively. The SCPPM error correction is effective compared to the hard decision. The BER drops rapidly to zero as the photon intensity increases. By extracting the photon levels where ${\rm{BER}} \,< {10}^{-5}$ as the photon number thresholds, dependences of *IPN* thresholds and *DPN* thresholds at different ${R}_{\text{PPM}}$ can be obtained, as shown in Fig. 5c. Meanwhile, the system detection efficiency defined by the ratio between *DPN* and *IPN* is also plotted. Similar to the photon counting rates shown in Fig. 2a, the system detection efficiency reduces as ${R}_{\text{PPM}}$ increases. Therefore, the receiver requires a higher *IPN* to support error-free communication. *DPN* thresholds show less influence of ${R}_{\text{PPM}}$, and its slight increase is caused by the pile-up effect at high counting rates, which worsens the timing jitter and photon number resolving accuracy.

The sensitivity (in units of photons per bit) characterizes the minimum required photons per receiving one bit. As shown in Fig. 5d, this study demonstrates a communication receiver that has a sensitivity of 1.47 photons/bit at 480 Mbps, 1.76 photons/bit at 800 Mbps, and 3.40 photons/bit at 1.2 Gbps. Furthermore, by sacrificing some detection efficiency and increasing the PPM slot rate to 8 GHz, a 1.5 Gbps bit rate could be obtained with a sensitivity of 7.41 photons/bit. In comparison, The LLCD receiver has a sensitivity of 3.48 photons/bit at 622 Mbps^11^. This high bit rate performance of this study is also competitive with the DPSK coherent receiver (7.3 photons/bit at 1.244 Gbps) in the LCRD experiments^37,38^. Therefore, these results are encouraging and show that a communication system based on photon counting can also reach Gbps data rates with excellent sensitivity compared to communications based on coherent modulation and detection.

#### Communication at strong background noise

Typically, laser communications are preferred to be operated at night due to a clear background. Daylight operations are welcomed because the total communication duration can be increased. For certain missions where the transmitter can only be seen on Earth during daylight, particularly for solar system boundary exploration, daylight operations are necessary. However, the strong background noise from the daylight sky, which is prominent (for instance, 0.78 photons per PPM slot as calculated in^7^) compared with the signal photons received from very far away, prevents the operation of laser communication during the daytime. The presence of intense background noise leads to a significant increase in the BERs. To mitigate these effects, one possible approach is to incorporate a narrow-band filter or shift the communication wavelength outside the range of the background spectrum. Another potential strategy involves appropriately attenuating both signal photons and noise photons in the received signal^39^. In this study, we utilize 4-QD’s PNR capacity along with an error correction coding strategy to address this issue.

**Fig. 6:**
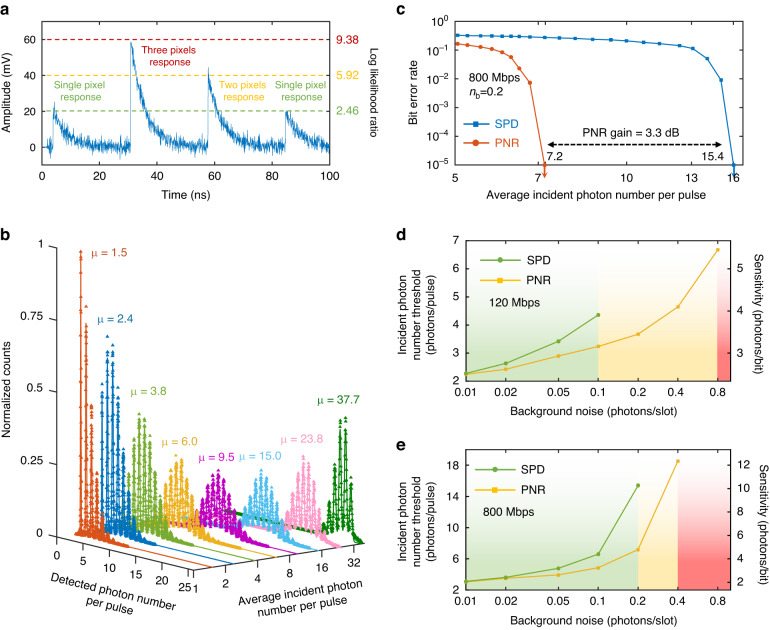
**Communications at strong background noise. a** Examples of output pulse waveforms for different photon number detections and corresponding *LLR* values. The estimated background noise photons are ${n}_{\text{b}}$ = 0.1 photons/slot. **b** Photon number distributions detected by the 4-QD at different incident photon intensities. A matched filter is applied to improve the discrimination between adjacent peaks. **c** Error correction performance comparison of FEC with and without PNR detections. The background noise photons are  0.2 photons/slot, and the bit rate is 800 Mbps. Dependences of incident photon number thresholds for FEC with and without PNR detections at bit rates of 120 Mbps (**d**) and 800 Mbps (**e**), respectively. The green area marks the range where the FEC can work for both SPD and PNR detectors, and the yellow area represents the range where the FEC can work for PNR detectors and not for SPD. The red area marks the range where FEC fails to work for two kinds of detectors

As mentioned above, the series nanowire architecture has a quasi-PNR capability. As shown in Fig. 6a, the output pulse height is proportional to the number of fired pixels, from which the Poisson photon-number distribution with a known spatial distribution of the incident light spot can be deduced. As shown in Fig. 6b, the sum of all four outputs gives a maximum photon number of 24. This is important since such PNR capability provides a dynamic range of 4.6 bits instead of 1 bit, bridging the gap between a single-photon detector and a linear photodetector. Therefore, for a soft-decision FEC, such as SCPPM, the detected photon number can convert into a more accurate LLR based on an estimate of the channel as follows:^32^

2 $${\rm{LLR}}={\log }_{2}\frac{p\left(k|1\right)}{p\left(k|0\right)}=k\,\cdot\, {\rm{lo}}{{\rm{g}}}_{2}\left(1+\frac{{n}_{{\rm{s}}}}{{n}_{{\rm{b}}}}\right)-{n}_{{\rm{s}}}$$

where *k*, *n*_s_, and *n*_b_ represent the detected photon number, the average signal photon number, and the average background noise photon number, respectively. As shown in Fig. 6c, compared to single-photon counting mode where *k* is set in a binary mode (*k* = 0 for no detection and *k* = 1 for any photon detections), a PNR capability gives a gain of 3.3 dB in the incident photon number threshold at a background noise of 0.2 photons/slot and bit rate of 800 Mbps.

Changing the background noise, the incident signal photon thresholds at two different bit rates of 120 Mbps and 800 Mbps were extracted. Results are shown in Fig. 6d, e. As the background noise increased, more errors were caused by noise photon detections. Therefore, a higher incident photon number was required for correcting errors. There was a maximum background noise level that the FEC could tolerate. The maximum noise levels for the single-photon detector were about 0.1 photons/slot at 120 Mbps and 0.2 photons/slot at 800 Mbps. However, the maximum noise levels for the PNR detector increased to about 0.8 photons/slot at 120 Mbps and 0.4 photons/slot at 800 Mbps, which was a large improvement.

#### Beam position sensing by the photon-counting 4-QD

Precise tracking the beam is the precondition for establishing a space-to-ground communication link. A 4-QD, which has a 2 × 2 array configuration, is a simple position sensor used widely. When the beam spot is scanned, the photon counting rates *CR*_1_, *CR*_2_, *CR*_3_, and *CR*_4_ from the four quadrants have clear position dependences. The maximum likelihood estimator of the incident flux intensity *Φ* is given by $\hat{\varPhi }$ = ${\text{h}}{v}\,\cdot\, {CR}$, where ${\rm{h}}{v}$ is the photon energy. Therefore, a basic 4-QD centroid algorithm by replacing the inputs to *CR*_1_–*CR*_4_ can be applied^40^, which are as follows:

3 $$\Delta x={k}_{x}\,\cdot\, \frac{({{CR}}_{1}+{{CR}}_{4})-({{CR}}_{2}+{{CR}}_{3})}{{{CR}}_{1}+{{CR}}_{2}+{{CR}}_{3}+{{CR}}_{4}}$$

4 $$\Delta y={k}_{y}\,\cdot\,\frac{({{CR}}_{1}+{{CR}}_{2})-({{CR}}_{3}+{{CR}}_{4})}{{{CR}}_{1}+{{CR}}_{2}+{{CR}}_{3}+{{CR}}_{4}}$$

where ${k}_{x}$ and ${k}_{y}$ are the coefficients for converting the counting rate differences into position offsets Δ$x$ and Δ$y$ from the centroid of the beam spot, respectively. Then, the estimated spot position can be obtained.

The position-sensing ability can be integrated simultaneously with the communication to avoid splitting the incident signal light into a second optical path, improving the sensitivity of the receiver at the system level. When the beam spot shifts outside the center of the detector, the counting rates vary significantly. Therefore, accurate focusing of the beam spot at the center can divide the incident photon flux evenly on every quadrant, maximizing the overall detection efficiency.

**Fig. 7:**
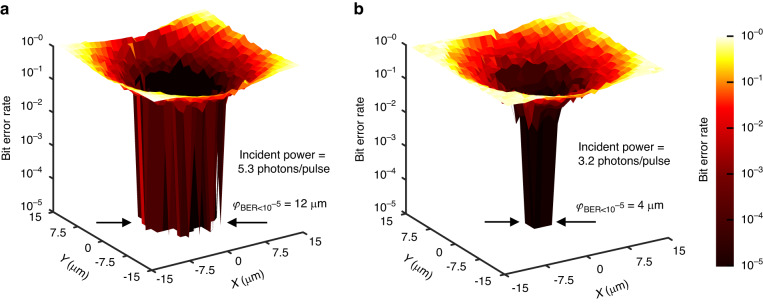
**Dependence of the BER on beam position**. Communication BER (at a bit rate of 800 Mbps) vs. position at incident photon numbers 5.3 photons/pulse (**a**) and 3.2 photons/pulse (**b**), respectively. The brighter color means a higher BER, and the darker color means a lower BER

The discussions above suggest that the communication BER is related to the beam position. As shown in Fig. 7a and b, the BER was measured by scanning the beam spot positions at two different incident photon intensities. The BER rolled down rapidly when the beam spot approached the center of the detector. Consequently, a spot shifting margin $\varphi$ below which the BER was less than $1\,\times \,{10}^{-5}$ was defined. $\varphi$ was 12 µm at the bit rate of 800 Mbps and *IPN* = 5.3 photons/slot. Considering the detector had a total detection area of 20 µm × 20 µm, it indicated that an error-free communication could be maintained until the laser beam was not shifted off the center by 60%. When the incident photon number reduced to 3.2 photons/pulse, $\varphi$ reduced to 4 µm. Therefore, these results prove that an accurate centering of the beam spot is necessary for obtaining the best sensitivity.

## Discussion

The experimental results show that the proposed detector architecture, as well as the associated signal processing method and algorithm, can address several challenges in space-to-ground laser communications. With a small number of output lines, bit rates from 120 Mbps to 1.5 Gbps have been demonstrated, covering a broad range for various space laser communication missions. Benefiting from the PNR capability, the receiver is robust to background noise and exhibits daylight operation potential. Meanwhile, the 4-QD architecture gives a position-sensing capability that stabilizes the receiver at its best sensitivity. Practical coupling from a large aperture telescope may want a larger detector. Thus, a tradeoff between the maximum bit rate and the active area appears, which needs further optimization or uses a superconducting microstrip detector^41–44^. Moreover, hardware implementation of signal processing as well as decoding is required for communicating in real-time. With these specialties and further developments, we envision that the receiver prototype will be installed in future space missions.

## Materials and methods

### Device details

The nanowire of the device was made from a 5 nm thick NbN film. NbN has lower kinetic inductivity and faster thermal cooling than amorphous films, such as tungsten-silicon (WSi) and molybdenum-silicon (MoSi)^45^. The array covered an area of 20 µm × 20 µm. The nanowire was 90 nm wide and 60 µm long for each sub-pixel, giving *L*_k_ = 52 nH. The shunt resistance was *R*_p_ = 50 Ω, pushing the reset time constant ${\tau }_{\text{f}}^{\text{f}}$ to 1 ns for each sub-pixel. This time constant was just above the latching limit to maximize the detection speed^46^. Shunt resistors were fabricated as close as possible to the sides of the nanowires to avoid any parasitic inductance from connection wires.

### Signal pre-processing steps

The 4-QD has four outputs *n* = 1–4. Each output pulse *r*_*n*_(*t*) is the superposition of the signal *s*_*n*_(*t*) and noise ${g}_{n}(t)$, which can be expressed as follows:

5 $${r}_{n}\left(t\right)={s}_{n}\left(t\right)+{g}_{n}\left(t\right)$$

The photon detection pulse *s*_*n*_(*t*) can be expressed by a double exponential function as follows:

6 $${s}_{n}\left(t\right)=A\,\cdot\, ({{\rm{e}}}^{-\frac{t}{{\tau }_{1}}}-{{\rm{e}}}^{-\frac{t}{{\tau }_{2}}})$$

where, ${\tau }_{1}$ and ${\tau }_{2}$ represent the rising and falling times of the pulse, respectively. $A$ represents the pulse amplitude of the single photon response. We apply a matched filter to maximize the signal-to-noise ratio of which the details are described in our previous study^33^. *y*_*n*_(*t*) is the waveform after the matched filter, which can be written as follows:

7 $${y}_{n}\left(t\right)={r}_{n}\left(t\right)\otimes {s}_{f}({t}_{0}-t)$$

where $\otimes$ denotes the convolution operation. However, the pulses are still indistinguishable after the matched filter, so a deconvolution method is needed to identify their positions and heights. The deconvolution template $D(t)$ is as follows:

8 $$D\left(t\right)={s}_{f}\left(t\right)\otimes {s}_{f}({t}_{0}-t)$$

Then, the deconvoluted signal $V(t)$ is as follows:

9 $$V\left(t\right)={y}_{n}\left(t\right)\odot {D}(t)$$

where $\odot$ denotes the deconvolution operation. The final step is to find the peak locations and heights of $V\left(t\right)$, marking the photon arrival times and detected photon numbers, respectively.

The complexity associated with signal processing and coding poses a significant concern in hardware implementation. We are currently engaging in addressing this issue. The entire process can be executed on a field programmable gate array board with fast analog-to-digital converters for digitizing and processing detection pulses in real-time. Hardware implementation can leverage techniques from advanced wireless communication systems, while efficient algorithms should be optimized.

### Supplementary information

Supplementary information for A compact multi-pixel superconducting nanowire single-photon detector array supporting gigabit space-to-ground communications
