# Supplementary material for: A compact multi-pixel superconducting nanowire single-photon detector array supporting gigabit space-to-ground communications
Source: Light Sci Appl. 2024 Jan 22;13:25. doi: 10.1038/s41377-023-01374-1 (PMC10803749; doi:10.1038/s41377-023-01374-1)
Supplement: Supplementary file 1 — Supplementary information for A compact multi-pixel superconducting nanowire single-photon detector array supporting gigabit space-to-ground communications [file 41377_2023_1374_MOESM1_ESM.pdf]

Supplementary Information for

# A compact multi-pixel superconducting nanowire single-photon detector array supporting gigabit space-to-ground communications

## Author Information

HAO HAO,[1] QING-YUAN ZHAO,[1,2]✉ YANG-HUI HUANG,[1] JIE DENG,[1] FAN YANG,[1] SAI-YING RU,[1] ZHEN LIU,[1] CHAO WAN,[2] HAO LIU,[2] ZHI-JIAN LI,[2] HUA-BING WANG,[1,2] XUE-COU TU,[1,3] LA-BAO ZHANG,[1,3] XIAO-QING JIA,[1,3] XING-LONG WU,[4] JIAN CHEN,[1,2] LIN KANG,[1,3] AND PEI-HENG WU[1,3]

[1]*Research Institute of Superconductor Electronics (RISE), School of Electronic Science and Engineering, Nanjing University, Nanjing, Jiangsu 210023, China*

[2]*Purple Mountain Laboratories, Nanjing, Jiangsu 211111, China*

[3]*Hefei National Laboratory, Hefei, Anhui 230088, China*

[4]*National Laboratory of Solid State Microstructures and Department of Physics, Nanjing University, Nanjing 210023, China;*

✉ qyzhao@nju.edu.cn

# 1. Device fabrication

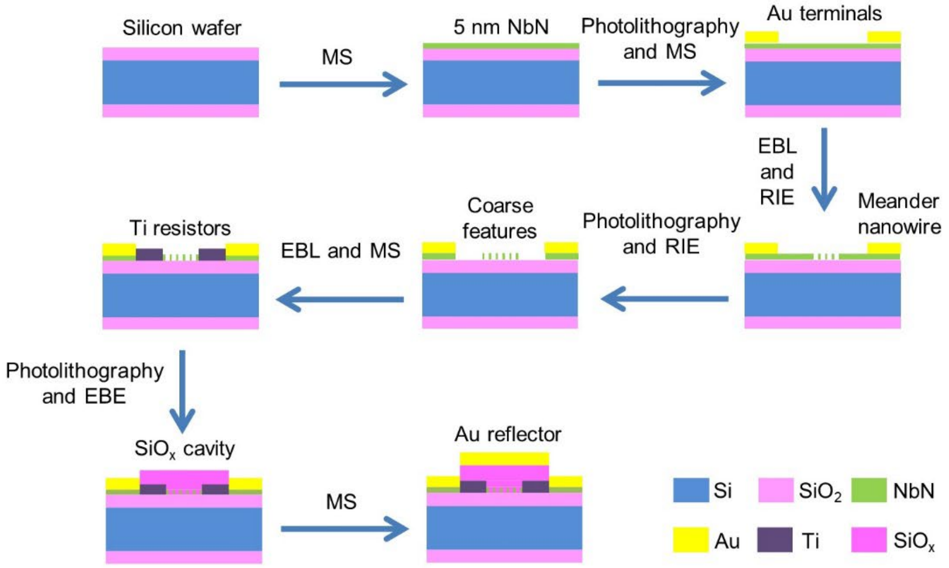


**Fig. S1. Fabrication processes of the four-quadrant detector.**

The fabrication processes of the 4-QD are shown in Fig. S1. The substrate was a silicon wafer with silicon dioxide on both sides. The thickness of the silicon wafer was 300 μm, and the silicon dioxide was 268 nm. The niobium nitride (NbN) superconducting film was deposited by magnetron sputtering (MS). The gas ratio during sputtering was $Ar:N_2 = 7.5:1$. The deposition rate was 1.3 $nmsec^{-1}$. The thickness of the NbN film was about 5 nm, which gave a sheet resistance of 290 $\Omega square^{-1}$ and a critical superconducting transition temperature of 7 K. After the film deposition, the contact pads and the alignment marks were patterned by photolithography and a following sputtering of Ti/Au. The next step was fabricating the meander nanowire. The total area of the 4-QD was 20 μm × 20 μm. The width of the nanowire was 90 nm, and the filling factor of the nanowire was $3^{-1}$. Electron-beam lithography was used to pattern the nanowire. The electron-beam resist was PMMA A4. Resist patterns were transferred to the film by Reactive Ion Etching (RIE). We used SF6 and CHF3 as reactive gases to etch the NbN film. After etching, the chips were immersed in N-methyl-2 at 80 °C for 20 mins to remove the residual PMMA. After this step, another photolithography made the line that connected the contact pads to the detector. The next step was to fabricate the on-chip Ti resistors which were made by the e-beam lithography and the MS. The sheet resistance of the Ti film was 16.4 $\Omega square^{-1}$. Before depositing the Ti film, ion beam milling was carried out to remove the residual resist and the oxide layer to get better ohmic contact. The last step was to fabricate the optical

cavity. The $SiO_x$ was used as the interlayer material for the optical cavity, which was deposited by electron beam evaporation (EBE). The thickness of $SiO_x$ was 272 nm. 10 nm Ti and 150 nm Au were then deposited on the top of $SiO_x$ as a reflector by MS.

## 2. Circuit calculation of the serial SNSPD

When incident photons induce a transition of the nanowire from its superconducting state to the normal state, resulting in a significantly higher resistance denoted as $R_n >> R_p$ (where $R_n$ represents the resistance of the normal state nanowire and $R_p$ is the parallel resistance), all inductances within the circuit can be disregarded. This is due to their relatively slow dynamic processes compared to the rapid dynamics occurring on the detector during this short period, with their impedance $j\omega L_k$ also exceeding that of the parallel resistor $R_p$. At this stage, the response detector can be approximated as a step current source (assuming the detector is operating in latching mode) depicted in Fig. S2**a**. Focusing solely on the fast-switching response of the photon-triggered nanowire, the signal at this moment represents a high-frequency signal, where $j\omega L_k$ significantly surpasses $R_p$ enabling us to approximate the circuit as Fig. S2**b**. The output current of the circuit can be determined using Eq. 1, while Eq. 2 allows for calculating the output voltage.

$$I_o = (I_B - I_f)/(n + R_L/R_p) \quad \text{(Eq.1)}$$

$$V_o = (I_B - I_f) \cdot R_p/(1 + n \cdot R_p/R_L) \quad \text{(Eq.2)}$$

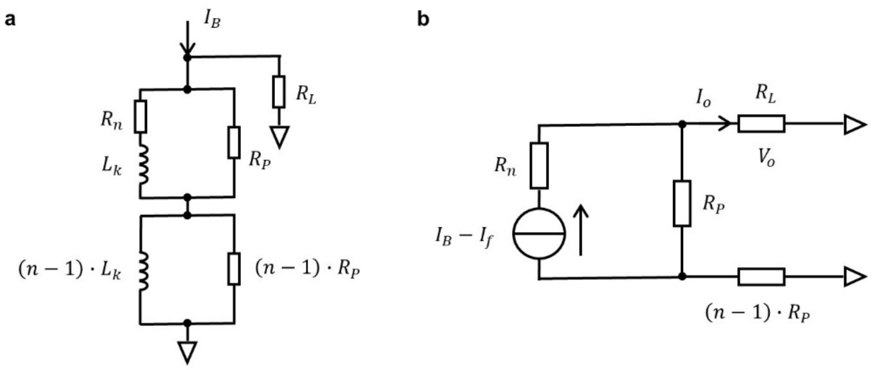


**Fig. S2. Equivalent circuit diagram of a series SNSPD array for single-photon detections.**

## 3. Instruments and components used in the measurement setup

In this section, we will present an overview of the essential instruments and components comprising the measuring setup.

The cryogenic amplifier utilized in this system is the CITLF1 which is positioned at 4.2K. It is biased to have a gain of 35 dB at 1 GHz. The implementation of cryogenic amplifiers aims to enhance the signal-to-noise ratio (SNR) of the output signal from the serial SNSPDs.

The high-speed arbitrary waveform generator (AWG) utilized in this system is the M8195A model from Keysight, which is equipped with an M9502A case. This AWG features four output channels and achieves a maximum sampling rate of 65 $GSasec^{-1}$ (with marker). It has been employed to convert the encoded data into high-speed electrical signals.

The electro-optical modulator (EOM) utilized in this system is the MXER-LN-20 manufactured by iXblue. It is equipped with a pulse RF amplifier (DR-PL-20-MO) and a DC bias controller (MBC-DG-LAB-A1). This EOM possesses a bandwidth of 20 GHz and an extinction ratio of 40 dB at a wavelength of 1550 nm. It converts high-speed electrical pulses into optical pulses.

The cryogenic nanopositioning system utilized Attocube's ANPx101 and ANPz101 nanopositioners, along with an ANC350 controller. To achieve triaxial XYZ movement, three nanopositioners are employed, consisting of two ANPx101s and one ANPz101. The nanopositioners have been used to couple the incident spot on the detector accurately.

The real-time oscilloscope utilized in this system is the HDO9404-MS manufactured by LeCroy. It has a bandwidth of 4 GHz and a sampling rate of 40 $GSasec^{-1}$. It has been employed for measuring the output pulse of the detector and recording the output signals during the communication experiment.

Two laser sources are utilized in the experiment. The first one is a TSL-710 continuous laser source from Santec, featuring an adjustable wavelength range of 1480 to 1640 nm and a maximum output power of 13 dBm. The second source is a femtosecond pulsed laser (FPL-M2CFF model from Calmar Laser) operating at a central wavelength of 1550 nm with pulse widths below 0.5 ps and a repetition rate of 20 MHz. The continuous laser served as the EOM source for generating high-speed optical signals during the communication experiment, while the femtosecond laser was employed to assess detector performance parameters such as timing jitter.

A cryostat designed by Cryomech is utilized to achieve the required operation temperature for the SNSPD. Throughout the experiments, this cryostat has maintained a temperature of 1.51 K and 1.80 K with a cooling capacity of 30 mW and 300 mW respectively.

## 4. Timing jitter

The timing jitter test system setup is almost the same as Fig. 3**a** in the main article, except a femtosecond laser with a repetition rate of 20 MHz was used as the laser source. The synchronization signal of the laser source and the output signal of the detectors were all read by the oscilloscope, and the time delay of the two signals was measured. All four quadrants were biased at 90% $I_c$. The time delay statistics of each four quadrants are shown in Fig. S3. The Gaussian distribution was used to fit the results, and the full width at half maximum (FWHM) of the distribution shows the timing jitter. The 3rd and 4th quadrants' jitters are higher than those of the 1st and 2nd quadrants, which was due to the differences in the readout circuits and cryo-amplifier. The cryo-amplifiers of the 3rd and 4th quadrants had worse SNR and made the timing jitter increase.

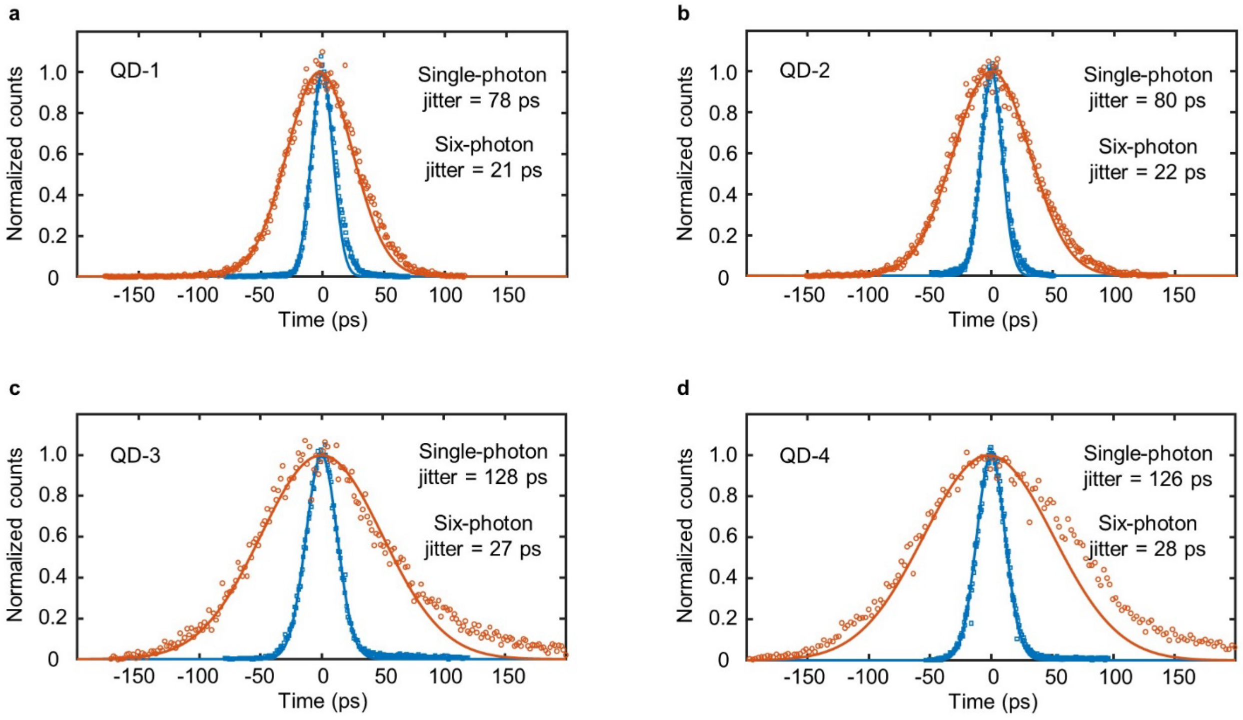


**Fig. S3: Timing jitters of the detector's all four quadrants.**

## 5. Device detection efficiency

The device detection efficiency has been measured in the experiment setup shown in Fig. S4**a**. A continuous laser with adjustable wavelength was used as the source. Due to the detector used in this work being polarization-sensitive, a polarization controller was used the optimize the laser polarization state. As shown in Fig. S4**b**, by adjusting the polarization state, the maximum count rate of the detector was 1.7 times higher than the minimum. Then the laser was split into two parts by a beam splitter, while one was read by a power meter, and another was attenuated by two attenuators. Next, the laser was connected to a collimator and then went in the cryostat through the window and three filters. The window was mounted at 300 K and the filters were mounted at 4.2 K. The filters used in the communication experimental setup and the device detection efficiency test setup were different. The commercial filters used in the communication experimental setup had a relatively lower total transmission efficiency which was 81.7% as described in the main article. The custom-made filters used in this device detection efficiency test had a particularly high transmission efficiency at the wavelength of 1550 nm as discussed below. The transmission of the window at 1550 nm was 99.8%. The transmission at room temperature of the filters is shown in Fig. S4**d**. The first filter was a narrow-band filter of 1550 nm which had a bandwidth of 12 nm, and the other two filters were wide-band filters which were used for suppression of background noise. The total transmission of this set of filters at room temperature was 95.6%. The detector's spectrum at 4.2 K is shown in Fig. S4**e**. The overall spectrum of the filters at 4.2 K had a shift of about 5 nm compared to that at room temperature, while the bandwidth maintained 12 nm. The oscillation of the spectrum was due to the detector's substrate rather than the filters. The Gauss spot emitted from the collimator had a diameter of 1242 μm (4σ), and the active area (except the corner parts which could absorb the light but could not respond) of one quadrant was 9 μm × 9.2 μm. Finally, 0.013% of the spot energy would be incident on the detector. As shown in Fig. S4**c**, an optical cavity was designed to achieve higher optical absorptivity, which had a simulated absorption of 93.6% at 1550 nm. As a result, excluding the optical coupling loss and filters' transmission, the detection efficiencies of each quadrant were 89.4%, 91.0%, 92.6%, and 93.4% respectively. The total detector's efficiency is the average of the four quadrants which is 91.6%.

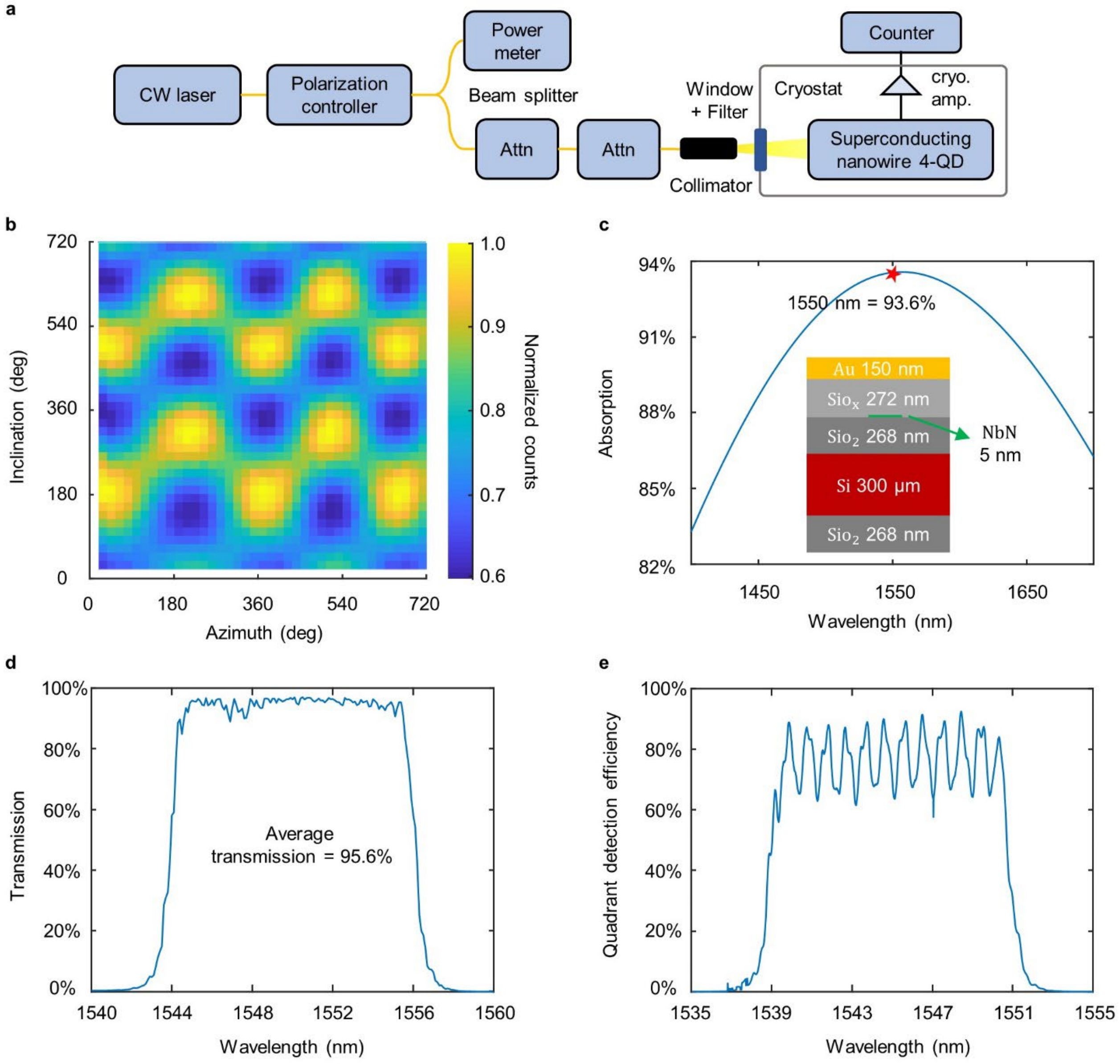


**Fig. S4: Characterizations of the detection efficiency. a.** Experiment system setup for detection efficiency measurement. **b.** The detector counts at different polarization states. **c.** Simulated absorption of the optical cavity. **d.** The transmission of the filters at room temperature. **e**. Response spectrum of the first quadrant, measured at 12.6 μA bias current.

# 6. Communication system setup and spot coupling

In the communication system, the laser signal from the collimator passed through the window and filters and then was focused by a focusing lens. The lens was mounted on an XYZ-nanopositioner. By changing the distance between the lens and the detector, the size of the spot could be adjusted. Each quadrant would have different count rates when the spot moved at the detector's surface. As shown in Fig. S5**a**, four figures correspond to the four

quadrants' count rates, and each quadrant achieved the maximum count rate at different spot positions (only if the spot hit on the center of the quadrant, could this quadrant obtain a maximum count rate). To get the maximum detection efficiency of the whole detector, the spot should be moved to the center of the detector. As shown in Fig. S5**b**, this is the total count rate of four quadrants. The detector achieved a maximum system detection efficiency of 72.7% with a spot diameter of 17.8 μm ($1/e^2$ width). During the communication experiments, to utilize the full performance of the detector, the diameter of the light spot was increased to 29.6 μm, and the system detection efficiency was reduced to 52.5%.

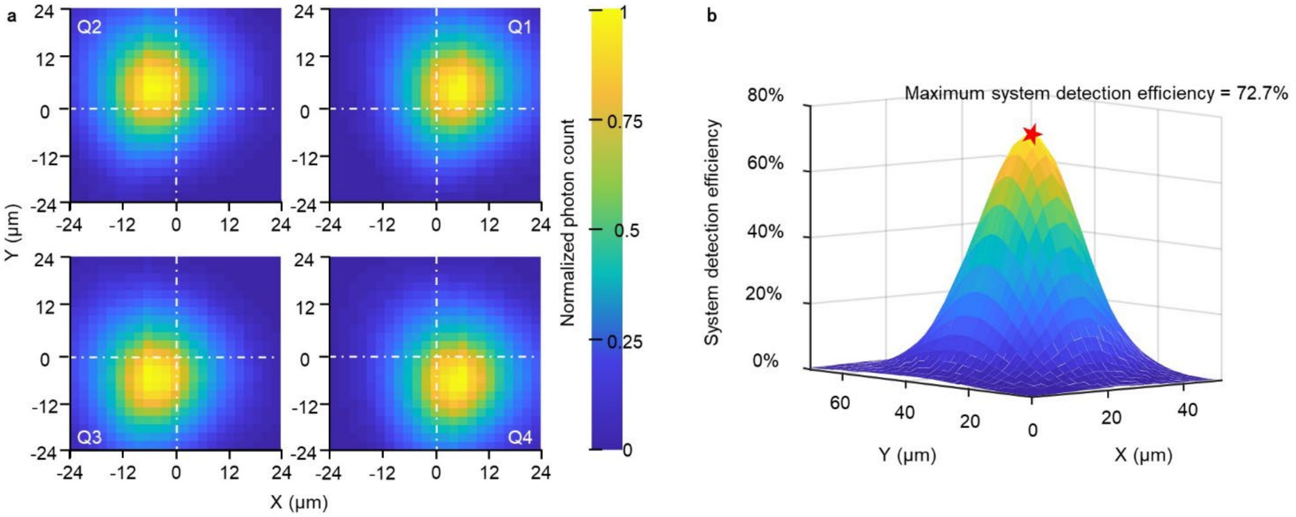


**Fig. S5: Detection efficiency with different spot positions.**

## 7. Optimization of the order of PPM modulation

The communication rate of PPM modulation, $R_{\mathrm{PPM}}$, is determined by its slot time $T_{\mathrm{S}}$, dead time $T_{\mathrm{D}}$, modulation order $M$, and the error correction coding rate $R$ (which is fixed at 1/2), as indicated in Eq.3. The slot time and dead time of PPM are limited by the timing jitter and reset time of the detector. Consequently, $R_{\mathrm{PPM}}$ can be computed using the corresponding parameters of the detector for different modulation orders, as illustrated in Fig. S6.

$$R_{\mathrm{PPM}} = R\frac{log_2 M}{M \cdot T_{\mathrm{S}} + T_{\mathrm{D}}} \qquad \text{(Eq.3)}$$

Different from a single SNSPD, which requires a recovery time to return to a superconducting state, the 4-QD detector in this study comprised 24 sub-pixels in total, thereby reducing the dead time by counting photons parallelly. Thereby, in our experiments, $T_{\mathrm{D}}$ was set to be 0 ns. The detector's minimum single photon timing jitter was 78 ps. $T_{\mathrm{S}}$ was set to be 125 ps, which was about two times wider than the timing jitter for reducing the error

at the high counting rate.

Although the detector's minimum timing jitter could achieve 78 ps, in the case of high communication rates, we must widen the PPM slot time to ensure error-free code communication (125ps) due to issues such as output pulse pile-up and degraded SNR.

With a $T_D$ of 0 ns and a $T_S$ of 125 ps, as depicted by the blue line in Fig. S6, the PPM is capable of achieving a maximum data rate of 4 Gbitsec$^{-1}$ at an order of $M = 4$. However, in our experiment, we observed that the detector's recovery time was insufficient short to maintain error-free communication at this order. Therefore, we had two options: either introducing a dead time while keeping the modulation order at 4 (as shown by the red line in Fig. S6) or increasing the modulation order to 8 (with a higher modulation order, each symbol could keep a longer time to make the detector recover). Both methods successfully achieved error-free communication experimentally. However, the latter combination offered a higher data rate of 1.5 Gbits$^{-1}$. Thus, we chose $M = 8$ and $T_D = 0$ ns in our communication experiments.

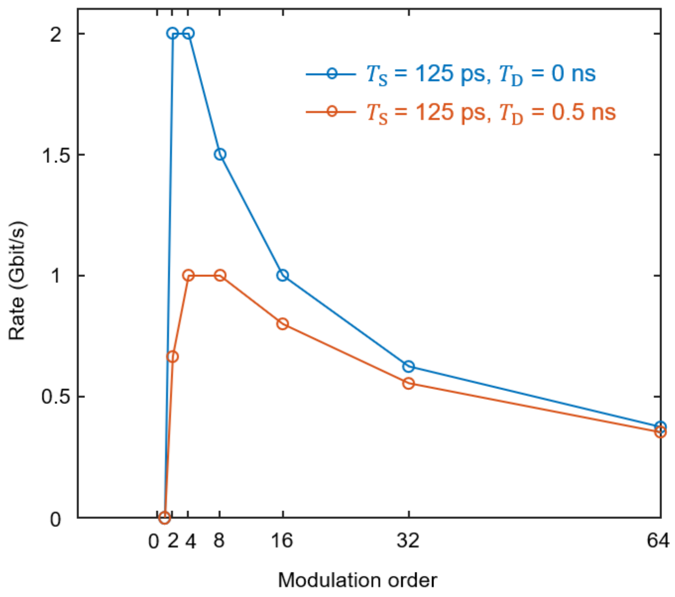


**Fig. S6: The maximum PPM communication rate at different modulation order**

## 8. Configurations of the PPM at different data rates

To generate the PPM formatted optical pulses, we used a high-speed arbitrary waveform generator whose sample rate is 65 GSasec$^{-1}$ to generate electrical pulses, which were then input to a fast electro-optical modulator. In our

communication experiments, we used 8-PPM. Considering the timing jitter from detectors, the optical pulse width was narrower than the slot width. Details configurations of the optical pulse width, slot width, and symbol width (8 times of the slot width) are given in **Table S1**.

**Table S1. Configurations of the PPM at different data rates**

| **Bit rate**<br>(including 1/2 code rate) | **Optical pulse width**<br>(sample points) | **Slot width**<br>(sample points) | **Symbol width**<br>(sample points) |
|---|---|---|---|
| 120 Mbps | 312.5 ps (20) | 1.56 ns (100) | 12.5 ns (800) |
| 240 Mbps | 156.25 ps (10) | 781.25 ps (50) | 6.25 ns (400) |
| 480 Mbps | 125 ps (8) | 390.63 ps (25) | 3.13 ns (200) |
| 0.8 Gbps | 93.75 ps (6) | 234.38 ps (15) | 1.88 ns (120) |
| 1.0 Gbps | 62.5 ps (4) | 187.5 ps (12) | 1.5 ns (96) |
| 1.2 Gbps | 46.88 ps (3) | 156.25 ps (10) | 1.25 ns (80) |
| 1.5 Gbps | 31.25 ps (2) | 125 ps (8) | 1 ns (64) |

## 9. List of Abbreviations

**Table S2. List of abbreviations**

| | | | |
|---|---|---|---|
| Lunar Laser Communication Demonstration | LLCD | Forward error correction | FEC |
| Astronomical units | AU | Likelihood ratio | LLR |
| European Space Agency | ESA | Electro-optical modulator | EOM |
| Signal-to-noise ratio | SNR | Bit error rates | BER |
| Pulse position modulation | PPM | Incident photon number per pulse | IPN |
| Single-photon detectors | SPDs | Detected photon number per pulse | DPN |
| Superconducting nanowire single-photon detector | SNSPD | Niobium nitride | NbN |
| System detection efficiency | SDE | Tungsten-silicon | WSi |
| Four-quadrant | 4-QD | Molybdenum-silicon | MoSi |
| Photon number resolving | PNR | Scanning electron microscope | SEM |
